# Chicago Veterinary College

**Published:** 1896-10

**Authors:** 


### CHICAGO VETERINARY COLLEGE.

THIS college has, as heretofore announced, extended its course to one of three years of six months each. G. S. Baker, D.V.S, professor of special and comparative physiology and histology, retires from the corps of instructors. Olof Schwarzkopf, V.M.D., late dean of the McKillip Veterinary College and veterinarian to the Illinois Humane Society, will fill the professorship of gross pathology. F. H. Davis, M.D.C., succeeds A. McBride, M.D.C., as assistant to the chair of theory and practice. N. A. Graves, M.D., is added as assistant to the chair of chemistry.

